# Magnetic Field Modeling and Visualization of the Europa Clipper Spacecraft

**DOI:** 10.1007/s11214-023-00974-y

**Published:** 2023-05-26

**Authors:** Corey J. Cochrane, Neil Murphy, Carol A. Raymond, John B. Biersteker, Katherine Dang, Xianzhe Jia, Haje Korth, Pablo Narvaez, Jodie B. Ream, Benjamin P. Weiss

**Affiliations:** 1 Jet Propulsion Laboratory, California Institute of Technology, Pasadena, CA USA; 2 Department of Earth, Atmospheric, and Planetary Sciences, Massachusetts Institute of Technology, 77 Massachusetts Avenue, Cambridge, MA USA; 3 Department of Climate and Space Sciences and Engineering, University of Michigan, Ann Arbor, MI USA; 4 Applied Physics Laboratory, Johns Hopkins University, Laurel, MD USA

**Keywords:** Europa Clipper, Europa Clipper Magnetometer, Plasma Instrument for Magnetic Sounding, Gradiometry, Spacecraft magnetic field modeling

## Abstract

The goal of NASA’s Europa Clipper Mission is to investigate the habitability of the subsurface ocean within the Jovian moon Europa using a suite of ten investigations. The Europa Clipper Magnetometer (ECM) and Plasma Instrument for Magnetic Sounding (PIMS) investigations will be used in unison to characterize the thickness and electrical conductivity of Europa’s subsurface ocean and the thickness of the ice shell by sensing the induced magnetic field, driven by the strong time-varying magnetic field of the Jovian environment. However, these measurements will be obscured by the magnetic field originating from the Europa Clipper spacecraft. In this work, a magnetic field model of the Europa Clipper spacecraft is presented, characterized with over 260 individual magnetic sources comprising various ferromagnetic and soft-magnetic materials, compensation magnets, solenoids, and dynamic electrical currents flowing within the spacecraft. This model is used to evaluate the magnetic field at arbitrary points around the spacecraft, notably at the locations of the three fluxgate magnetometer sensors and four Faraday cups which make up ECM and PIMS, respectively. The model is also used to evaluate the magnetic field uncertainty at these locations via a Monte Carlo approach. Furthermore, both linear and non-linear gradiometry fitting methods are presented to demonstrate the ability to reliably disentangle the spacecraft field from the ambient using an array of three fluxgate magnetometer sensors mounted along an 8.5-meter (m) long boom. The method is also shown to be useful for optimizing the locations of the magnetometer sensors along the boom. Finally, we illustrate how the model can be used to visualize the magnetic field lines of the spacecraft, thus providing very insightful information for each investigation.

**Supplementary Information:**

The online version contains supplementary material available at 10.1007/s11214-023-00974-y.

## Introduction and Motivation

Europa Clipper is set to launch in October 2024 and will perform Mars and Earth gravity assist maneuvers prior to entry into the Jovian system in 2030 where multiple flybys of Europa (Bradley et al. 2022) will be performed to investigate the properties of its subsurface ocean (e.g. thickness and electrical conductivity) using magnetic induction. The Europa Clipper Magnetometer (ECM) investigation is the primary science instrument for measuring this phenomenon, and is composed of three three-axis fluxgate (FG) sensors mounted on an 8.5-m long boom (Kivelson et al. 2023). The ECM fluxgate magnetometers operate by driving a ferromagnetic ring core into and out of saturation using a primary set of coils which modulates the ambient magnetic field intended to be measured, while a secondary set of coils is used to sense the effect. The second harmonic of the response is demodulated in firmware and used as a control signal to null the field around the core using a third set of coils, where the feedback current is a proportional measure of the field. Using three sensors serves three major purposes for this investigation: (1) it facilitates measurement of the spacecraft field gradient along the boom, which enables its dipolar and quadrupolar (i.e. dipole center) character to be characterized and removed, (2) it provides added margin to the ECM error budget compared to a two-sensor configuration, and (3) it provides redundancy with gradual loss to science objectives in the event of sensor failures. The Plasma Instrument for Magnetic Sounding (PIMS), composed of 2 sensors, each equipped with two Faraday cups (FC), will be measuring the plasma density, temperature, and velocity. Together with state-of-the-art magnetohydrodynamic models, PIMS will aid in determining the plasma-induced magnetic fields that arise from Jupiter’s co-rotating plasma currents and Europa’s ionospheric currents (Westlake et al. 2023). Because each of these instruments is susceptible to the local magnetic field, stray fields originating from the spacecraft can obscure the measurements of each instrument if they are large enough. Additionally, because a 1.5 nanotesla (nT) precision requirement is imposed on the magnetic field measured at the two main frequencies of the system – the 11.2-hour Jupiter synodic period and the 84.5-hour orbital period of Europa – in order to characterize the ocean thickness and electrical conductivity to within a 50% uncertainty – the spacecraft magnetic field must be characterized on the ground and in-flight so that its effects can be accounted for and removed from the measurements (see Kivelson et al. 2023).

There is a long history of NASA missions involving magnetic field measurements that span the Earth, planetary, and heliophysics disciplines. In each case, the presence of a magnetometer on a mission payload imposes additional constraints on the host spacecraft that are intended to reduce or mitigate spacecraft magnetic fields observed at the locations of the magnetometer sensors. These design constraints are often embodied in a magnetic cleanliness program which almost always consists of a boom that enables sensors to be placed away from the spacecraft bus where the spacecraft magnetic field is weaker. The requirements for such magnetometer booms have been the subject of much study, with theoretical treatments such as those of Ness et al. (1971) and Neubauer (1975). An overarching conclusion has been that a spacecraft should carry at least two magnetometer sensors at different positions along the boom to adequately remove spacecraft field contributions from the ambient field intended to be measured. Examples of the two-sensor approach to specific missions include: Ness et al. (1974) for Mariner 10, Behannon et al. (1977) and Ness et al. (1986, 1989, 1979) for Voyagers 1 and 2, Carr et al. (2005) and Georgescu et al. (2008) for Double Star, and Primdahl et al. (2006) for Ørsted. Neubauer (1975) has also considered a 3+ sensor approach to gradiometry.

**Fig. 1:**
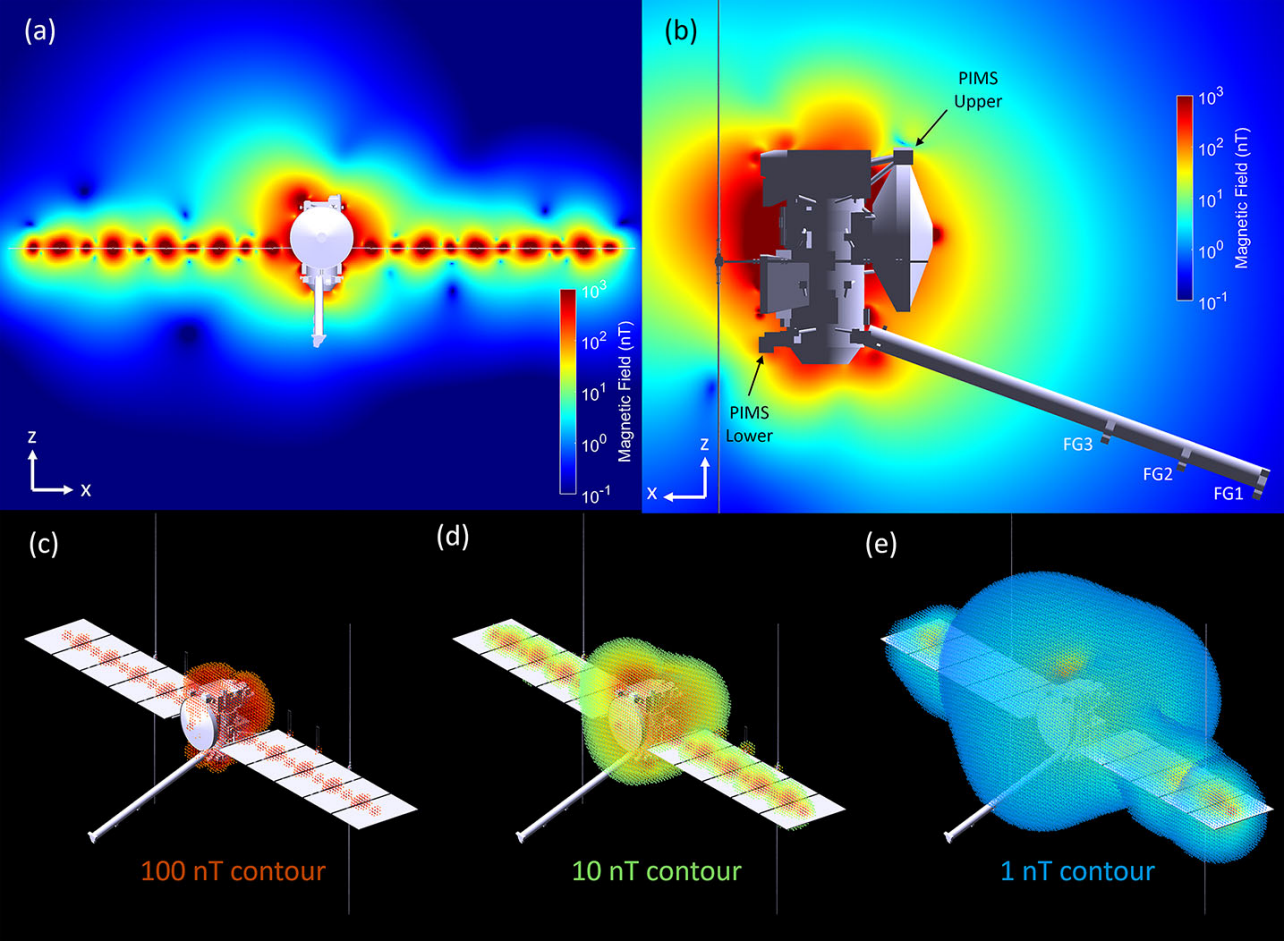
The DC spatial magnetic field magnitude of Europa Clipper Spacecraft. (**a**)–(**b**) Magnetic field magnitude of the spacecraft for two different cross-sectional views. (**c**)–(**e**) 3D magnetic field magnitude contours for field levels of 100 nT, 10 nT, and 1 nT, respectively

**Fig. 2:**
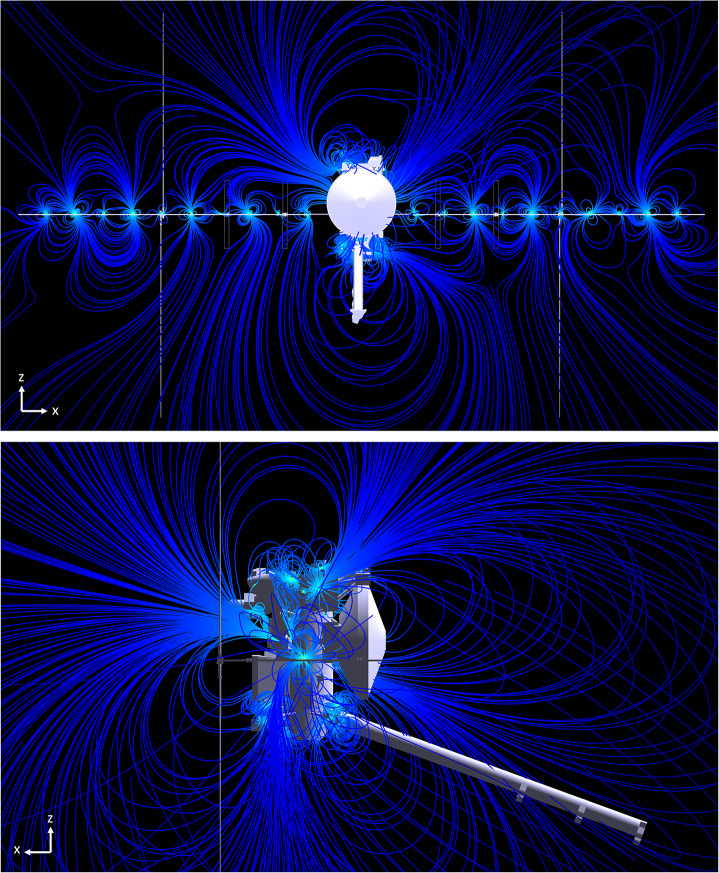
Representative magnetic field lines of Europa Clipper spacecraft in a zero-field environment for two different views

Here we extend this previous work to accommodate a three-sensor configuration to characterize and remove the complex magnetic field of the Europa Clipper spacecraft (Figs. 1, 2). The spacecraft is very large compared to the magnetometer boom (8.5-m long boom vs 30-m wide spacecraft when solar panels are deployed), and has large and spatially distributed field sources making a conventional two-sensor system not as effective for the needs of the science investigations. We represent the multipolar Europa Clipper spacecraft magnetic field with a model composed of over 260 magnetic dipole point sources. The accuracy of this multiple dipole approach has been validated for previous missions (Mehlem 1978a,b; Mehlem and Wiegand 2010; Neubauer and Schatten 1974; Zhang et al. 2007, Weiss et al. 2023). Each of these dipole moments has been extracted from magnetic field measurements made by the Europa Clipper project, provided by vendor document specifications or subject matter experts, or inferred from heritage part designs. We use this point-source magnetic moment model to assess the magnetic field at any point in space around the Europa Clipper spacecraft. We then visualize the magnetic field lines associated with the entire spacecraft as a system. These field line visualizations provide valuable information as to the orientation (field line direction) and strength of the magnetic field (color) at the sensor locations and also insight into the components on the spacecraft that are *magnetically-connected* to the sensors. We also show, via a Monte Carlo approach, how the uncertainty in amplitude and orientation of the magnetic moments that have not yet been measured translates into magnetic field uncertainties at the ECM and PIMS sensor locations. We then present a three-sensor gradiometry approach (using linear and non-linear methods) and show that it is capable of extracting the ambient field in the presence of the complex, but radially-decaying contaminating spacecraft field, and also describe how this performance metric can be used to optimize the location of the three sensor locations along the 8.5-m boom. This spacecraft magnetic field model will be coarsely validated during the assembly, test and launch operations (ATLO). However, the true magnetic signature of the spacecraft can only be realized in flight as it is challenging to characterize on the ground due to the presence of stray laboratory fields, Earth’s magnetic field, material thermomagnetic discrepancies, and restrictions on boom and solar array deployments and subsystems/instrument power-on states. Therefore, multiple characterization campaigns are scheduled after launch during the 5.5-year cruise to the Jovian system.

## Subsystem/Component Dipole Modeling

As it is essentially impossible to accurately measure the entire spacecraft magnetic field in the flight configuration on the ground, modeling, simulation, and testing are the only ways to ensure the magnitude of the spacecraft field is at or below its required levels at the positions of the magnetometer sensors and Faraday cups. To accomplish this, a magnetic moment is first allocated to all potential DC and AC current sources and magnetic materials on the spacecraft, arising from the various subsystems, instruments, and magnetic mechanical structures. This task is part of the magnetic cleanliness control program developed by the Electromagnetic-Interference (EMI) and Electromagnetic-Compatibility (EMC) team at JPL (Boghosian et al. 2013). For ECM, a maximum DC magnetic field of 1 nT is allowed at the outboard sensor. (This limit was derived from an analysis that demonstrated sufficient removal of the spacecraft magnetic field using the three-sensor approach assuming large sensor offset uncertainty.) ECM also has a series of AC requirements, shown in Table 1, designed to prevent interference with the operation of the instrument (e.g. sensor drive and second harmonic demodulation frequency). For PIMS, a maximum DC magnetic field of 250 nT is allowed at each of the FC sensors. For the AC peak-to-peak field, the levels should be < 125 nT in the frequency range of 100 Hz to 500 Hz, due to the instrument’s frequency and phase detection scheme tuned to 300 Hz.

**Table 1:** DC and AC spacecraft magnetic field requirements imposed at the ECM and PIMS sensor locations

Sensor	DC	AC	AC Frequency Range	AC Frequency Range Notes
	(nT)	**A**_p-p_ (pT)	(Hz)	
ECM FG1 (outboard sensor)	< 1	< 280	0.0001 < f ≤ 0.05	Solar panel rotation and illumination variation rate
*Note:* the two inboard sensors FG2 and FG3 do not have associated magnetic field requirements		< 100	0.05 < f ≤ 5	Common AC spacecraft-fields
		< 50	5 < f ≤ 5,000	Common AC spacecraft-fields
		< 500	5,000 < f ≤ 20,000	Sensor drive and second harmonic frequencies
		< 5,030	20,000 < f ≤ 100,000	Variations above 20 kHz are not significant
PIMS Upper and Lower	< 250	< 125,000	100 < f < 500	Instrument modulation band

The EMI/EMC magnetic cleanliness team ensures that spacecraft fields are kept to a minimum by verifying that all components, subsystems, and instruments are designed and built with good magnetic cleanliness practices and conform to assigned subsystem level magnetic moments. This includes the use of nonmagnetic materials wherever possible, applying best-practices in cable harness wiring (e.g., twisted-pairs throughout with minimum loop areas and solar array back-wiring to reduce the induced magnetic fields associated with large current loop sources), and magnetic moment self-canceling orientations, to effectively null large-scale residual fields. Each source is independently measured by the EMI/EMC team with multiple test magnetometers positioned around the component so that a dipole magnetic moment can be determined. Each magnetic source is represented by a magnetic moment magnitude, orientation, and position on the spacecraft.

Throughout the build process of the spacecraft on the ground, the EMI/EMC team uses the model to help monitor the aggregate magnetic field at the ECM FG sensors and PIMS FCs and to determine the available mitigation options when large magnetic field sources are encountered (Narvaez 2018). Based on prior measurements, some of the magnetic field sources that can be problematic include the four reaction wheel assemblies (RWAs) that orient the spacecraft, the radio-frequency (RF) switches and traveling-wave tube amplifier (TWTA) from the telecommunication subsystem, the power control and distribution assembly (PCDA), the solar array (SA) panels, and the three batteries that power the spacecraft. If necessary, the team counters the effect of these large magnetic sources, which often are sometimes unavoidable in the design, by using compensation magnets or high-permeability shielding (e.g., mu-metal), all of which all are also included in the model.

In order to ensure requirements are met, the spacecraft magnetic field model captures the worst-case scenario (i.e., increased allocations for margin, magnetic materials assumed to be in a high magnetized state, current loop sources in high power consuming mode, etc.) since not all dipole magnetic moments from component measurements are readily available during the build process and the orientations of the component’s dipole moments in spacecraft coordinates are usually unknown. At times, the model not only includes the associated magnetic moment extracted from component measurements but also from vendor provided document specifications, subject matter expert estimates from analyses, and heritage design inferences. These estimates include added margin and are built into the spacecraft magnetic field model. To enhance the accuracy of this worst-case modeling and to ensure ECM and PIMS science objectives can be met, spacecraft level magnetic field modeling, visualizations, and Monte Carlo simulations are conducted as discussed in the following sections.

## Spacecraft Level Magnetic Field Modeling

As noted previously, due to the large (∼ 30 m) span of the deployed solar panels and the inability to characterize the spacecraft deployment configuration on the ground, simulation is required to ensure that the magnetic field at the senor locations or any points around the spacecraft are within tolerable limits. The magnetic field of the spacecraft, $\boldsymbol{B}_{\mathrm{SC}} (\boldsymbol{r})$, can be estimated at position $\boldsymbol{r}$ by summing the individual fields from $N$ individual magnetic moments $M_{i}$, each located at position $\boldsymbol{r}_{M_{i}}$:

1 $$ \boldsymbol{B}_{\mathrm{SC}} (\boldsymbol{r})= \frac{\mu _{0}}{4\pi} \sum _{i=1}^{N} \frac{3 \left [ \left ( \boldsymbol{r} - \boldsymbol{r}_{M_{i}} \right ) \boldsymbol{\cdot} \boldsymbol{M}_{i} \right ] \left ( \boldsymbol{r} - \boldsymbol{r}_{M_{i}} \right ) - \boldsymbol{M}_{i} \left \vert \boldsymbol{r} - \boldsymbol{r}_{M_{i}} \right \vert ^{2}}{\left \vert \boldsymbol{r} - \boldsymbol{r}_{M_{i}} \right \vert ^{5}}. $$

Note that this equation is valid for both DC magnetic field evaluation and the AC field describing moments that exhibit a time dependence, i.e., $\boldsymbol{M}_{i} ( t )$. By taking the Fourier transform of this time dependent field $\boldsymbol{B}_{\mathrm{SC}} (\boldsymbol{r},t)$, one can assess the AC magnetic field at the sensor locations for any bandwidth of interest. For example, we have utilized this approach to estimate the time-dependent magnetic field from the solar panels which exhibit a magnetic moment that is constantly changing in both orientation and magnitude due to solar array rotations and variations in solar illumination angle, respectively.

Of particular interest is the magnitude of the magnetic field around the spacecraft. Using the magnetic moment model and Eq. (1), an estimate of the field can be computed on a grid of points around the spacecraft. Figure 1 (a) and (b) illustrate the spatial distribution of the magnetic field magnitude of two two-dimensional (2D) cross sectional views of the spacecraft. As illustrated, the boom is positioned to minimize the effects of magnetic sources emanating from the spacecraft. Figure 1 (c) and (d) illustrate the three-dimensional (3D) contours for three different magnetic field levels, 100 nT, 10 nT, and 1 nT, respectively.

## Spacecraft Magnetic Field Line Visualization

Another informative visualization that can be realized with this magnetic moment model is the magnetic field lines of the spacecraft, representing effective magnetic signature of the 260 sources on the spacecraft. Here, we implement a magnetic field line tracing routine based on the Euler method, also referred to as the first-order Runge-Kutta method. Improved accuracy can be obtained by using higher order midpoint approaches (e.g., 2nd or 4th order Runge-Kutta methods) (Press et al. 2007). However, the additional cost of computation that accompanies these higher order methods can be mostly avoided with the Euler method by introducing a smaller spatial step size.

**Fig. 3:**
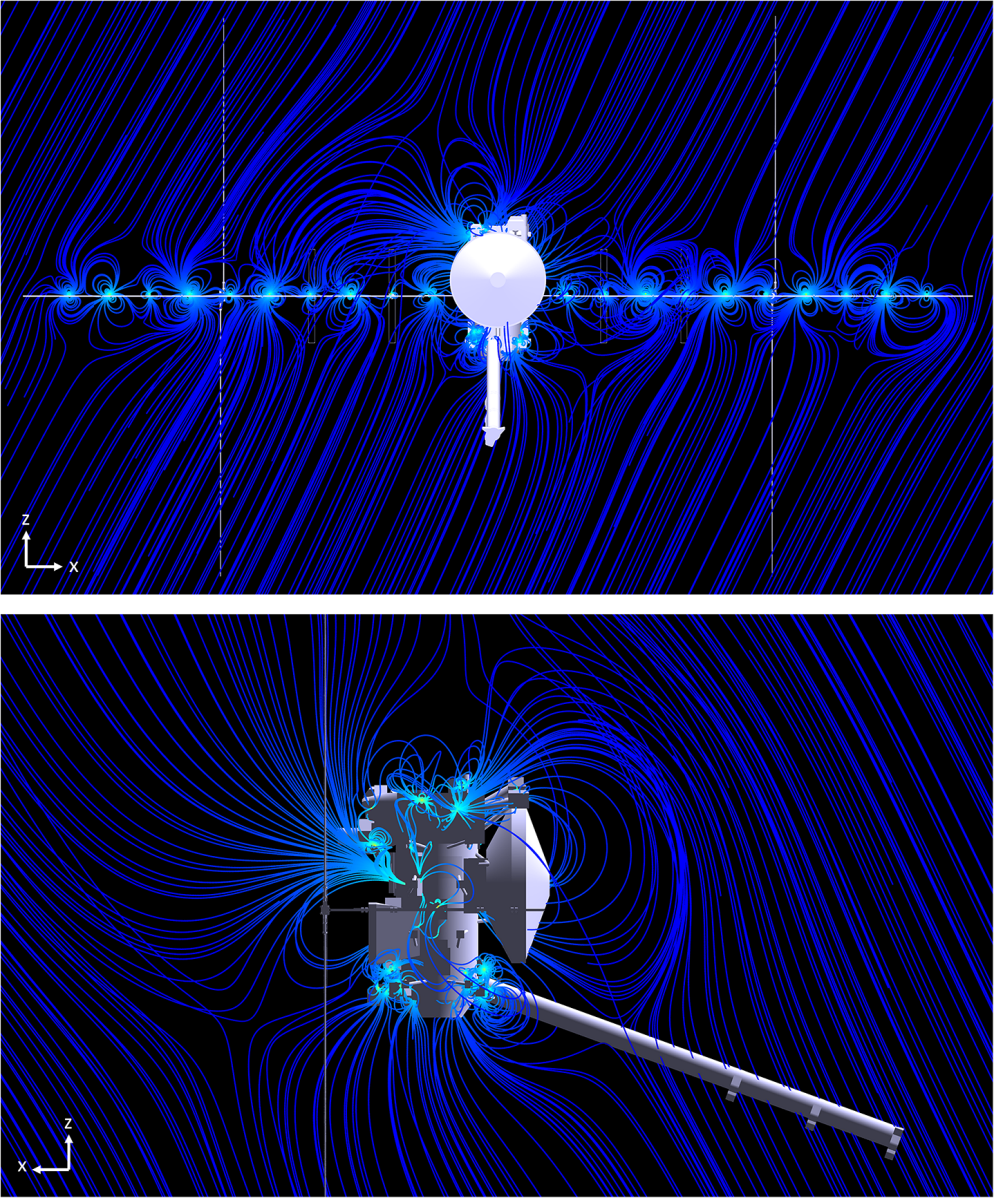
Representative magnetic field lines of Europa Clipper spacecraft in a low magnetic field environment (e.g., 5 nT) IMF, for two different views

Figures 2 and 3 illustrate two different cross-sectional views of two representations of the magnetic field lines associated with the Clipper spacecraft field, (1) in a zero-field and (2) non-zero field environment, respectively. For the latter, the introduced uniform ambient magnetic field has a magnitude of nearly 5 nT ($B_{x}$ = $B_{y}$ = 2 nT, $B_{z}$ = 4 nT), consistent with the typical IMF strength at 1 AU. Links to the downloadable animations of these spacecraft field line visuals, generated from a slightly older model, are provided in the appendix which gives a better sense of the 3D nature of the field lines. This approached was also used to simulate the field lines of the Psyche spacecraft (Weiss et al. 2023).

## Addressing Magnetic Field Uncertainty via Monte Carlo Simulations

As of the writing of this manuscript, there are still 211 magnetic moments of 260 that have not been fully characterized and or not yet had their final orientations defined. For all moments that are categorized as the latter, a magnetic field uncertainty of a factor of two is introduced as the strength of the magnetic field is two times stronger at the pole than at the magnetic equator for a dipole source. Therefore, in order to estimate the range of magnetic field from all sources on the spacecraft in arbitrary orientations, a Monte Carlo simulation was performed which entailed randomizing the orientation of those components that have not been measured and or those that are expected to be reoriented with respect to the magnetometer sensors (e.g., AC field sources such as magnetic materials and current sources on the solar panels). Randomizing the magnitude of the magnetic moment is also a possibility but the model already captures a worst-case upper limit for the magnetic moments of all unmeasured components.

**Fig. 4:**
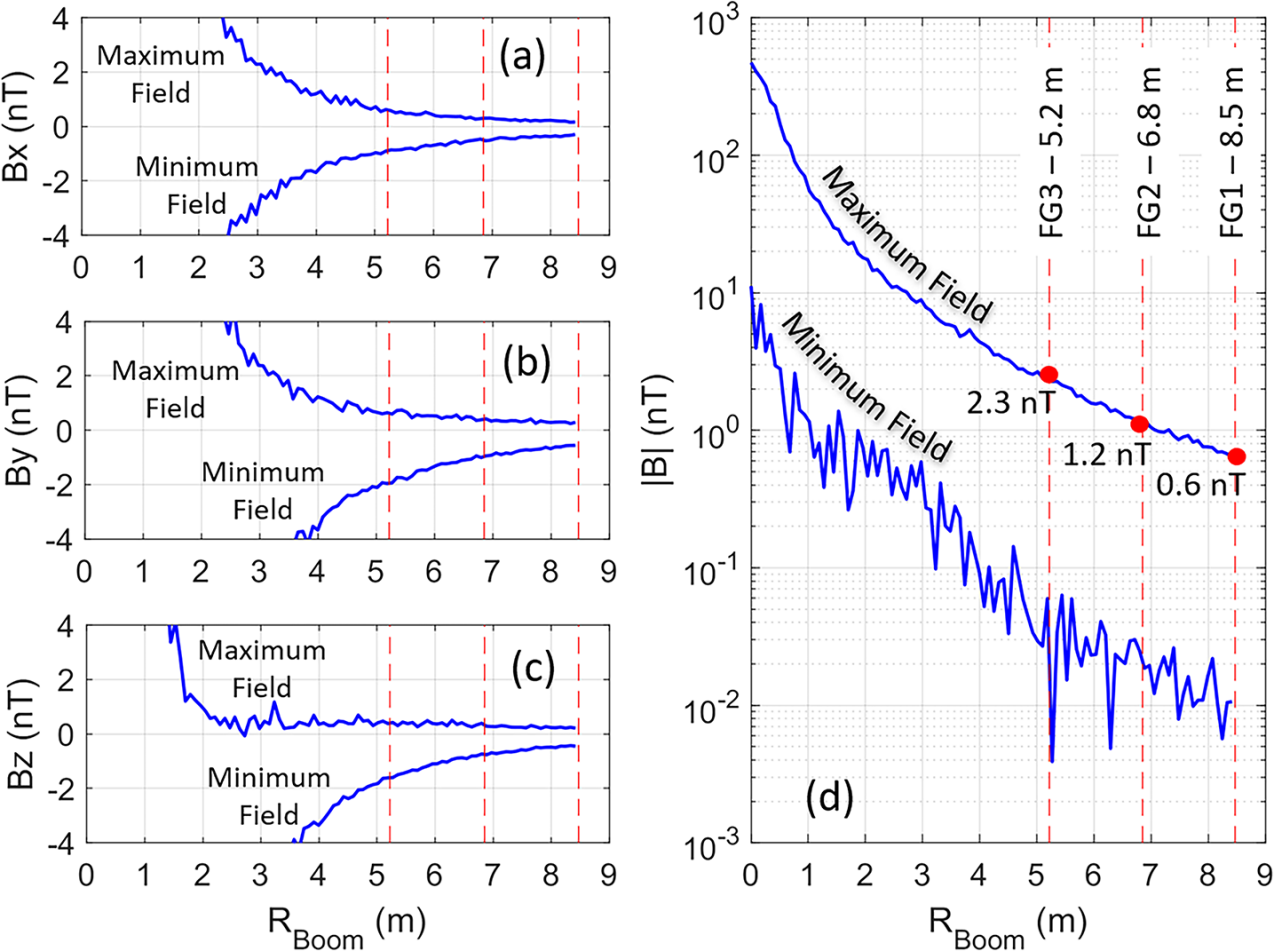
Spacecraft magnetic field along the boom. Maximum and minimum magnetic field calculated along the boom when the orientations of the unmeasured and non-static magnetic moments were randomized for the (**a**) Bx component, (**b**) By component, (**c**) Bz component, and (**d**) the magnitude |B|. The red vertical dotted lines in each panel represent the locations of the magnetometer sensors along the boom, those being 5.2 m (inboard), 6.8 m (middle), and 8.5 m (outboard) from the base

Figure 4 illustrates the results from such a simulation of the magnetic field. The magnitude and location of each magnetic moment identified on the spacecraft were fixed to a not-to-exceed allocation, but the moment orientation was allowed to vary for those moments not yet measured and those expected to have AC field character. The orientations of these magnetic sources were randomized 100,000 times for 100 possible discrete locations along the 8.5-m long boom. The moment azimuth direction $\phi $ was varied on the interval $U(-\pi ,\pi )$ and the moment elevation was varied $z\rightarrow U \left ( -1,1 \right )$ where the elevation angle was computed by $\theta = \cos^{-1} (z)$, thus providing uniform and sufficient variation of orientation to produce a meaningful estimate of the range of constructive and destructive interference patterns of the magnetic field sources for all locations along the boom. The individual panels show the maximum and minimum fields for each magnetic field component (i.e., $B_{x}$, $B_{y}$, $B_{z}$) along the boom (left three panels), along with the field magnitude plotted in log scale (right panel). The three red dotted lines shown in each panel represent the final magnetometer sensor positions located at 5.2 m, 6.8 m, and 8.5 m. Note that the determination of these locations is not arbitrary and is explained in Sect. 6.

**Fig. 5:**
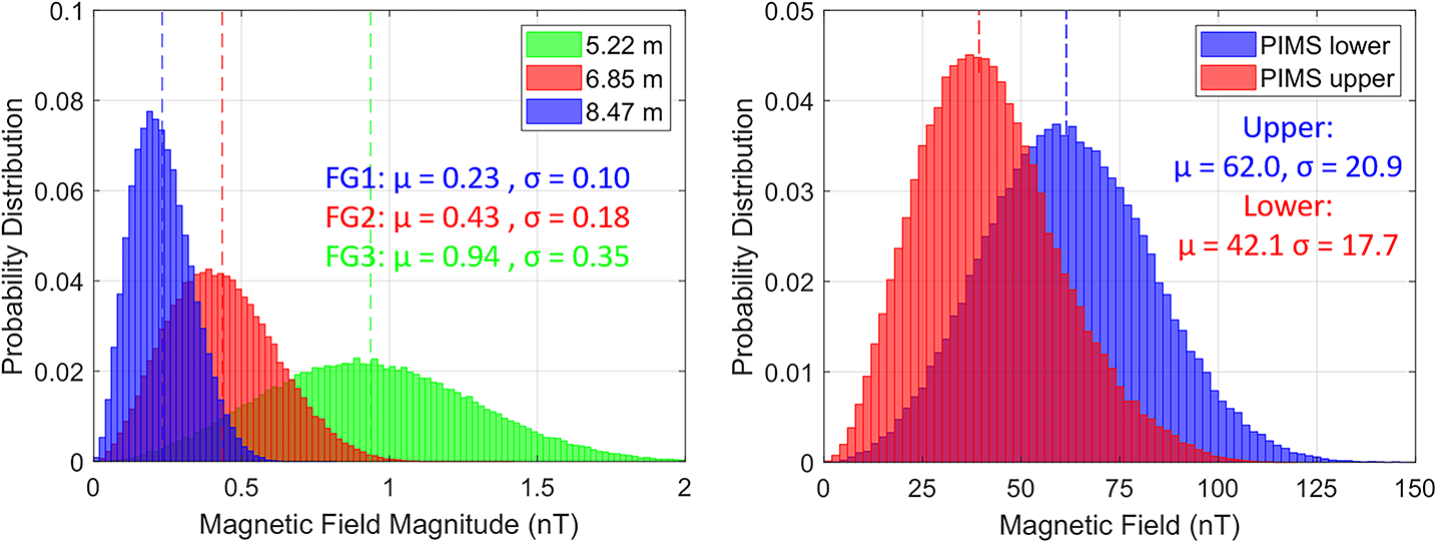
Magnetic field magnitude distributions acquired via Monte Carlo simulations that entailed randomly varying the orientation of magnetic moments associated with all magnetic sources on the Europa Clipper spacecraft at the positions of the (left) three ECM sensors and the (right) two PIMS sensors. The mean ($\mu $) and standard deviation ($\sigma $) is shown for each distribution. The dashed vertical lines highlight the mean of each distribution

Figure 5 highlights the results of two additional Monte Carlo simulations which illustrate the magnetic field distributions evaluated at: (left) the three FG sensor positions and (right) PIMS upper and lower FC sensor locations. For the outboard FG magnetometer sensor (FG1), the average field is roughly 0.23 nT, which extends to 0.53 nT when considering the 3$\sigma $ spread. This field value is lower than the 1 nT requirement with nearly 0.5 nT of margin. Similarly, the magnetic field mean for PIMS upper and PIMS lower is 62 nT and 42 nT, respectively. Inclusion of the 3$\sigma $ spread of the field results in a 125 nT and 95 nT field at the sensor locations, thus lower than the 250 nT magnetic field requirement imposed at each FC sensor.

## Spacecraft Magnetic Field Removal

Vector magnetic field measurements provided by the three, three-axis FG magnetometers will enable the characterization and removal of the stray magnetic field contribution from the Europa Clipper spacecraft and will provide redundancy in the event of sensor failure. Here, we describe the gradiometry approach that will be used by the investigation to disentangle the spacecraft field and the ambient field measured by each of the three FG sensors.

The magnetic field measurements $\boldsymbol{B}_{j}$ acquired from sensor $j$ include contributions from the ambient magnetic field vector $\boldsymbol{B}_{a}$ (assumed to be uniform across all three sensors), the contaminant spacecraft magnetic field $\boldsymbol{B}_{\mathit{SC},j}$, instrument noise $\boldsymbol{B}_{n,j}$, and instrument offsets $\boldsymbol{B}_{o,j}$,

2 $$ \boldsymbol{B}_{j} = \boldsymbol{B}_{a} + \boldsymbol{B}_{\mathit{SC},j} + \boldsymbol{B}_{n,j} + \boldsymbol{B}_{o,j}. $$

A full spherical harmonic estimate of the spacecraft field $\tilde{\boldsymbol{B}}_{\mathit{SC},j}$ at sensor $j$ can be realized by,

3 $$ \tilde{\boldsymbol{B}}_{\mathit{SC}, j} =- \nabla \sum _{l=1}^{N} R \left ( \frac{R}{r_{j}} \right )^{l+1} \sum _{m=0}^{l} \left \{ P_{l}^{m} \left ( \cos \theta _{j} \right ) \left [ g_{l}^{m} \cos \left ( m \phi _{j} \right ) + h_{l}^{m} \sin \left ( m \phi _{j} \right ) \right ] \right \}, $$

where $l$ and $m$ are the degree and order, $g_{l}^{m}$ and $h_{l}^{m}$ are the Gauss coefficients, $P_{l}^{m} \left ( \cos \theta \right )$ are the Schmidt semi-normalized Legendre polynomials, and $r_{j}$, $\theta _{j}$, and $\phi _{j}$ are the spatial coordinates (in the spacecraft frame) of the field at sensor $j$ in spherical coordinates. Prior to the 3-sensor magnetometer approach used by ECM, the Europa Clipper mission initially included a 4-sensor magnetometer investigation called the Interior Characterization of Europa using MAGnetometry (ICEMAG), which was descoped from the mission in 2019. The instrument comprised two fluxgate sensors and two scalar vector helium sensors which operate by exciting electron spin transitions within optically pumped ^4^He atoms. The four sensors produced a total of 14 measurements, a 3-axis vector measurement from each of the four sensors and two absolute scalar measurements from the two helium magnetometers. These measurements were sufficient in number to estimate the 3-element ambient magnetic field vector $\boldsymbol{B}_{a}$, in addition to the eight spherical harmonic parameters ($g_{1}^{0}$, $g_{1}^{1}$, $h_{1}^{1}$, $g_{2}^{0}$, $g_{2}^{1}$, $h_{2}^{1}$, $g_{2}^{2}$, $h_{2}^{2}$) required for a quadrupolar ($N=2$) expansion of Eq. (6). This configuration allowed for the use of a shorter boom (e.g., 5 m) for which the quadrupolar contribution of the spacecraft field is stronger, but could be characterized and removed using four sensors. Instead, the descope resulted in a three-fluxgate sensor solution, but required an 8.5-m long boom as the 3-sensor option provides only 9 measurements, which is insufficient to characterize the full quadrupole nature of the magnetic field close to the spacecraft. In this configuration, three parameters are needed to estimate the ambient field vector, $\tilde{\boldsymbol{B}}_{a}$, which leaves six free parameters to model the spacecraft field. These six available parameters are sufficient to estimate the spacecraft field $\tilde{\boldsymbol{B}}_{\mathit{SC},j}$ at sensor $j$, modeled as a single offset dipole with magnetic moment $\boldsymbol{M}_{\mathit{SC}}$ centered at location $\boldsymbol{r}_{\mathit{SC}}$,

4 $$ \tilde{\boldsymbol{B}}_{\mathit{SC}, j} = \frac{\mu _{0}}{4 \pi} \frac{3 \left [ ( \boldsymbol{r}_{j} - \boldsymbol{r}_{\mathit{SC}} )\boldsymbol{\cdot} \boldsymbol{M}_{\mathit{SC}} \right ] ( \boldsymbol{r}_{j} - \boldsymbol{r}_{\mathit{SC}} ) - \boldsymbol{M}_{\mathit{SC}} \left \vert \boldsymbol{r}_{j} - \boldsymbol{r}_{\mathit{SC}} \right \vert ^{2}}{\left \vert \boldsymbol{r}_{j} - \boldsymbol{r}_{\mathit{SC}} \right \vert ^{5}}. $$

The estimate of the total magnetic field $\tilde{\boldsymbol{B}}_{j}$, from sensor $j$, is computed by,

5 $$ \tilde{\boldsymbol{B}}_{j} = \tilde{\boldsymbol{B}}_{a} + \tilde{\boldsymbol{B}}_{\mathit{SC},j}. $$

Even though the spacecraft produces a highly multipolar field close to the spacecraft (with spherical harmonic content from $l=1$ to $\infty $, Hurwitz 1960), the offset dipole model provides a good approximation to the spacecraft magnetic field at the sensors, each in the far field. One way to solve for the 9 parameters contained in the three vectors $\boldsymbol{B}_{a}$, $\boldsymbol{M}_{\mathit{SC}}$ and $\boldsymbol{r}_{\mathit{SC}}$, is to minimize the sum of squared errors (i.e. L2-norm),

6 $$ \mathcal{E}= \sum _{j =1}^{3} \left \vert \tilde{\boldsymbol{B}}_{j} - \boldsymbol{B}_{j} \right \vert ^{2} $$

Note that other approaches can be used to solve this problem, including general relationships provided by Ness et al. (1971) and Neubauer (1975).

Two different methods can be used to perform this minimization. One can either (1) use a non-linear least squares approach (e.g., trust-region-reflective method, Coleman and Li 1994, 1996) to solve for $\boldsymbol{B}_{a}$, $\boldsymbol{M}_{\mathit{SC}}$, and $\boldsymbol{r}_{\mathit{SC}}$ directly or (2) fix $\boldsymbol{r}_{\mathit{SC}}$ and solve for $\boldsymbol{B}_{a}$ and $\boldsymbol{M}_{\mathit{SC}}$ using a more conventional linear-least squares approach. The former provides better accuracy in the near-field (i.e., closer to the spacecraft) at the cost of additional computation time and complexity, while the latter method is much faster and simpler at the cost of being less accurate at estimating the near field. However, with the current magnetic field model, the linear approach was shown to be just as accurate in the far-field where the sensors are located due to the more rapid fall-off of the higher order moments (e.g., the quadrupolar term falls off at 1/r^4^ and dipole only 1/r^3^), see results in subsequent section. In this work, to speed up computation time of the Monte Carlo simulations, we initially used the non-linear approach to solve for an average spacecraft dipole offset $\boldsymbol{r}_{\mathit{SC}}$ and then use it as the fixed value for the linear estimation approach. Coincidentally, the offset dipole inversion typically identified a dipole position near the base of the boom as producing the most accurate estimate of $\boldsymbol{B}_{a}$ and therefore was used in subsequent inversions of the fixed dipole approach.

## Optimizing FG Sensor Locations on the Boom

Each mission determines the sensor placement based on their science requirements. For two sensor systems, one sensor is placed as far from the spacecraft as possible and the second sensor is placed far enough from the prime sensor to observe a gradient in the spacecraft field. Ness et al. (1971) suggested placing the sensors so that ${r_{\mathit{out}}} / {r_{\mathit{in}}} =0.54$, with the ratios of the field at each sensor of approximately ${B_{\mathit{in}}} / {B_{\mathit{out}}} =6.5$. Placing the primary sensor at the tip of a long boom and the secondary sensor at the mid-way point on the boom satisfies this criterion. This approach was used by several missions including Mariner 10 (Giberson and Cunningham 1975), Cassini (Dougherty et al. 2004), and Juno (Connerney et al. 2017). If the spacecraft has not been through a strict magnetic cleanliness campaign, or the main function of the second sensor is for redundancy, it is preferable to place both sensors as far from the spacecraft as possible with the spacing determined by mechanical constraints and instrument crosstalk. Some missions that have used this approach are Mars Observer (Acuña et al. 1992), Rosetta (Glassmeier et al. 2007) and Double Star (Carr et al. 2005). A mixed approach to sensor placement has also been used based on the intended use for each of the magnetometers. The inboard sensor on Galileo was placed approximately 2/3 of the distance from the spacecraft as the outboard sensor. The justification was that the inboard sensor was intended to measure stronger fields so it could tolerate more spacecraft noise and the separation of the instruments allowed for gradiometry (Kivelson et al. 1992). In cases of shorter booms and more stringent magnetic cleanliness requirements, it is necessary to model sensor placement based on a set of worst-case magnetic field models and then determine how to fit the optimum position into the mechanical restraints imposed by the spacecraft. This is the approach used by both the Psyche and the Europa Clipper missions.

**Fig. 6:**
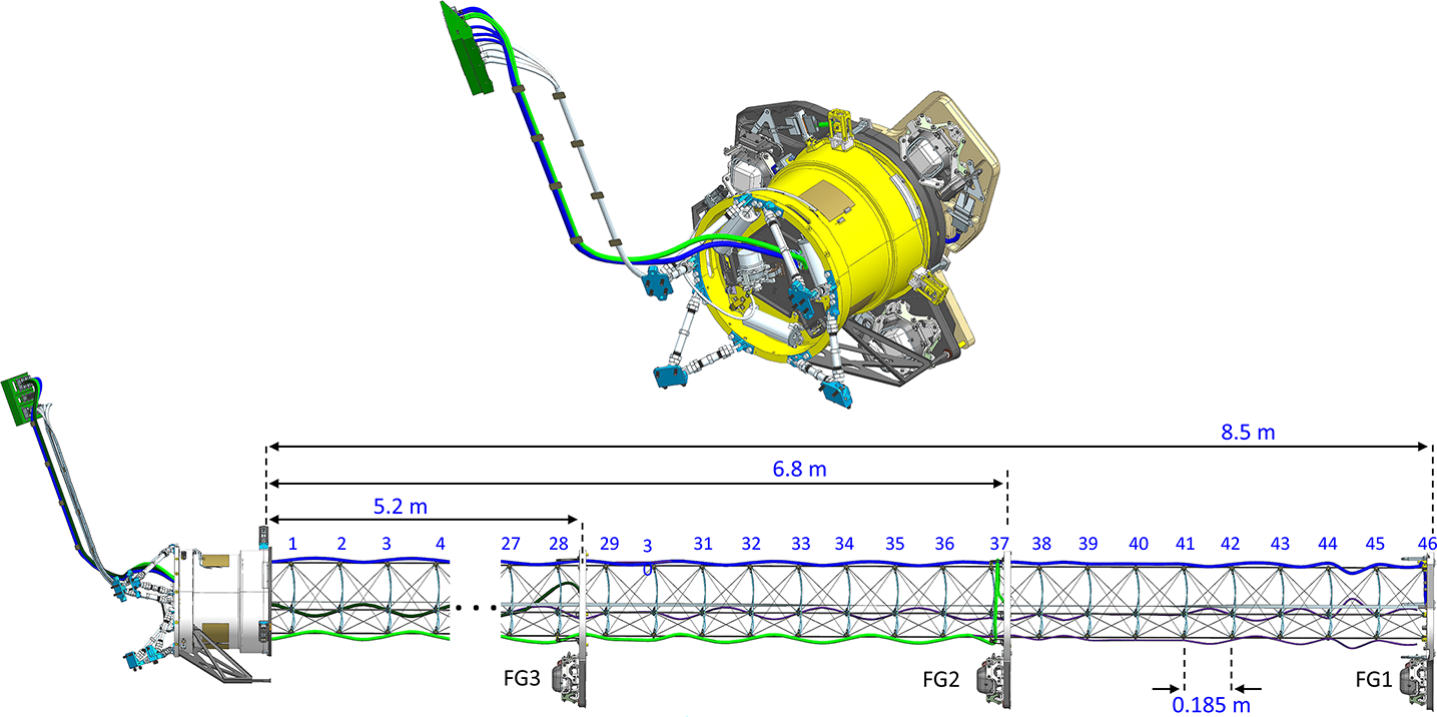
Magnetometer boom in (top) stowed and (bottom) deployed configuration

For ECM, the performance of the gradiometer is determined by varying the position of the two inboard sensors along the boom (the third sensor is ideally placed on the end of the boom). However, not all possible combinations of sensor positions are available because of mechanical constraints imposed by the structure of the coilable boom. More specifically, the sensors can be mounted only to relatively static end terminals at the end of each bay, see Fig. 6. The bays of the 8.5-m long coilable boom are spaced 0.185 m apart, providing a total of 46 discrete possible positions for sensor placement. To improve accuracy of the gradiometric measurement in light of each sensor’s measurement uncertainty and to minimize crosstalk between neighboring sensors, a minimum spacing of 0.7 m is required. Additionally, the three sensors must be positioned so that they do not physically interfere with one another when the boom is in its stowed or deploying configurations. This significantly limits the number of acceptable combinations of sensor configurations on the boom. One configuration that is ideal from a mechanical perspective is when the sensors are located at 5.2 m, 6.8 m, and 8.5 m from the base (see Fig. 6). What makes this sensor configuration especially attractive is that it not only optimally satisfies the mechanical constraints, but also provides acceptable (though not optimal) positions from a gradiometry perspective. In this configuration, the middle sensor is placed near the outboard sensor, thus providing redundancy in case of an outboard sensor failure.

In order to assess the optimal sensor positions along the boom and the expected errors of the gradiometry experiment with the mechanically-preferable configuration, we carried out a set of Monte Carlo simulations in which the locations of the middle and inboard sensors were varied along the boom (the outboard sensor remained fixed at the end of the boom) and the DC spacecraft field and the ambient magnetic field were estimated by the gradiometry method outlined previously. We carried out the analysis for a range of spacecraft magnetic field models and instrument noise patterns. (10,000 noise patterns and models were developed when linear methods were employed, but only 1,000 when non-linear methods were used). In our analysis, we simulated $\boldsymbol{B}_{j}$ at sensor $j$ as a superposition not only of the ambient field, $\boldsymbol{B}_{a}$ (assumed to be uniform across the flight system) and the spacecraft magnetic field, $\boldsymbol{B}_{\mathit{SC},j}$, but also the sensor and instrument noise, $\boldsymbol{B}_{n,j}$, and the sensor offset uncertainty, $\boldsymbol{B}_{o,j}$. In the simulation, components of the ambient field were sampled from a uniform distribution in the range $\pm 500\ \mathrm{nT}$. The noise patterns were formed by randomly sampling from a time-sequence generated from electronics white-noise of $50\ \mathrm{pT}/ \sqrt{\mathrm{Hz}}$ at $1 \mathrm{Hz}$ and sensor flicker-noise characterized by $100\ \mathrm{pT}/ \sqrt{\mathrm{Hz}}$ at $1 \mathrm{Hz}$, and the sensor offset uncertainty was randomly sampled from a uniform distribution in the range $0\ \mathrm{nT}$, $\pm 0.25\ \mathrm{nT}$, $\pm 0.5\ \mathrm{nT}$ or $\pm 1.0\ \mathrm{nT}$. (Note that the present measurements from the ECM magnetometer indicate flicker noise is dominant below 1 Hz and exhibits a white spectrum of < $20\ \mathrm{pT}/ \sqrt{\mathrm{Hz}}$ at frequencies above 1 Hz (Kivelson et al. 2023). Also, in-flight calibration routines will allow determination of offsets to within $\pm 0.5\ \mathrm{nT}$. Therefore, the simulations carried out here include the worst-case scenario for offset uncertainty determination. We estimated the expected offset knowledge for the ECM sensors from experience with the Analog Fluxgate Magnetometers (AFG) (Russell et al. 2016) of the Magnetospheric MultiScale (MMS) mission from which the ECM magnetometers derive heritage. Nearly four years of continuous measurements indicate that the MMS magnetometers have typical offsets ranging from +/- 7 nT per axis and with a long-term drift rate of 0.1 to 1 pT/day. If we assume the same performance as that provided by the AFG magnetometers on MMS, then we expect to have offset knowledge well below these values because of two types of planned ECM in-flight calibration activities using spacecraft rolls (Kepko et al. 1996) and properties of magnetic field Alfvénic fluctuations in the solar wind (Davis and Smith 1968; Belcher 1973; Acuña 2002; Leinweber et al. 2008).

**Fig. 7:**
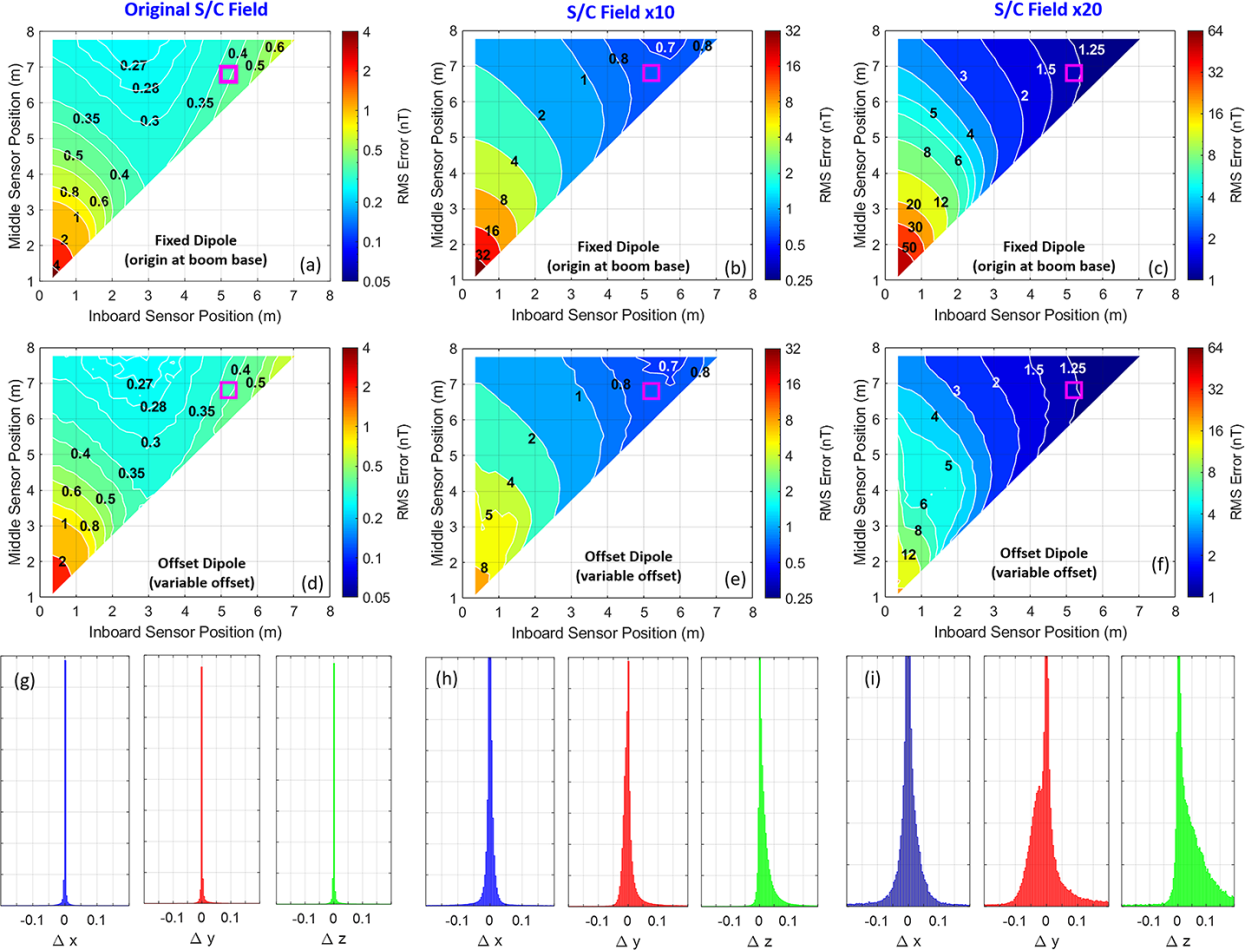
RMS contours of the estimated ambient field error for all possible positions of the inner and middle sensors along the boom (the outboard sensor is located at the end of the boom). The calculations include contributions from all noise sources (spacecraft field offsets, electronics white-noise, sensor flicker-noise, and sensor offsets) for an assumed offset uncertainty of +/- 0.5 nT. Panels (**a**) through (**c**) illustrate the results of the fixed dipole model approach assuming a dipole origin at the base of the boom for spacecraft field as-is (scale by 1), scaled by 10, and scaled by 20, respectively. Panels (**d**) through (**f**) illustrate the results of the non-linear modeling approach for the three different scalings of the spacecraft field where the dipole offset is solved for. Panels (**g**) through (**i**) illustrate the associated distributions of the positions of the estimated dipole offsets (in units of meters in spacecraft coordinates) with respect to the base of the boom for all Monte Carlo simulations shown in panels (**d**) through (**f**). In panels (**a**) through (**f**) of the figure, a magenta square has been drawn to illustrate the mechanically optimal configuration of the middle and inboard sensor for reference purposes

Figure 7 illustrates the differences between the results obtained from the linear approach and non-linear approach, described in the previous section, for three different scales of the spacecraft field (x1, x10, and x20 to illustrate extreme cases for uncertainty in spacecraft field), with the inclusion of instrument flicker noise and $\pm 0.5\ \mathrm{nT}$ offset uncertainty. As illustrated, the non-linear approach, which solves for the dipole offset, is optimal overall (i.e., results in the lowest RMS error of the ambient field estimate) when all sensor combinations are considered, especially when the spacecraft field is large and sensors are closer to the spacecraft. However, both methods perform almost identically for locations of the sensors in the far-field, near their mechanically optimal positions. Panels (g) – (i) of the figure provide insight as to why this is the case. In these panels, the distribution of the estimated dipole offset, with respect to the base of the boom (i.e., x = y = z = 0), is shown for each case of the spacecraft field scaling. For the current state of the spacecraft field (scale of x1), the estimated offset can be approximated to be more or less on the base of the boom and does not exhibit much spread in position. However, when the spacecraft field is scaled (i.e. x10 and x20), the spread of the offset distribution significantly increases and has more of a dramatic effect when modeling the estimated field. For example, note the large discrepancy between the (c) RMS error in the linear estimated ambient field and the (f) RMS error obtained in the non-linear approach, for sensor positions located near the spacecraft (within 5 m of the base of the boom). Here, the non-linear approach performs significantly better at removing the higher order quadrupolar nature of the magnetic field closer to the spacecraft (e.g., the ICEMAG regime), but is approximately the same in the far-field (e.g., the ECM regime). Therefore, we chose the much faster linear method to demonstrate the performance for subsequent tests assessing various levels of offset uncertainty.

**Fig. 8:**
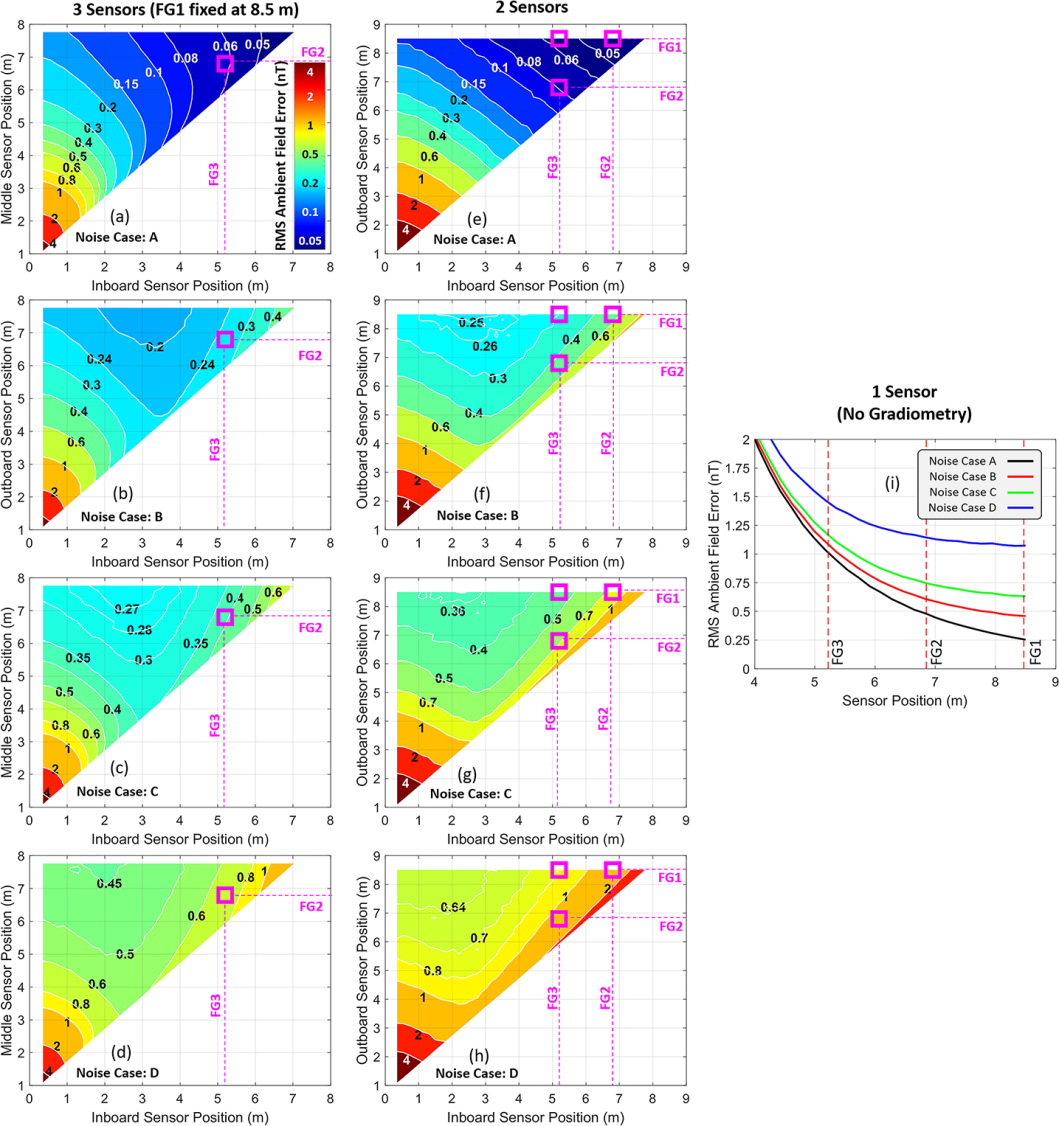
RMS contours of the estimated ambient magnetic field error for a range of positions of the sensors along the boom using a (left column) 3-sensor, (middle column) 2-sensor, and (right panel) 1-sensor (i.e. no gradiometry) approach. The residual errors include contributions from all noise sources (SC field offsets, electronics white-noise, sensor flicker-noise, and sensor offsets) for four different noise scenarios: Noise Case A: no instrument and sensor noise with no offset uncertainty, Noise Case B: instrument and sensor noise with ± 0.25 nT offset uncertainty, Noise Case C: instrument and sensor noise with ± 0.5 nT offset uncertainty, and Noise Case D: instrument and sensor noise with ± 1.0 nT offset uncertainty. In all panels of the figure, magenta squares have been drawn to illustrate the mechanically optimal and final configuration of the of the three FG sensors

Using the linear inversion method, additional Monte Carlo simulations were performed to gain a better understanding of how the sensor offset uncertainty affects the errors of the estimated ambient field using a 3-sensor, 2-sensor, and 1-sensor approach. The latter two schemes are included to illustrate the ambient field errors if 1 or 2 sensors happened to fail. These results are illustrated in Fig. 8 for four different instrument noise and offset scenarios. Noise case A entails no instrument and sensor noise with no offset uncertainty, noise case B entails instrument and sensor noise with +/- 0.25 nT offset uncertainty, noise case C entails instrument and sensor noise with +/- 0.5 nT offset uncertainty, and noise case D entails instrument and sensor noise with +/- 1.0 nT offset uncertainty. As illustrated in all of the datasets, larger offset uncertainty significantly increases the errors of the estimated ambient field and therefore should be kept as low as possible. Note also the improvement in performance that is achieved for each additional sensor that is used.

## Discussion

The results provided in the previous section collectively demonstrate that the optimal positions of the middle and inboard sensors are determined by a tradeoff between the intrinsic noise and offsets of the instruments, the higher-order moments of the spacecraft field, and the magnitude of the spacecraft field. High instrument noise and large offsets and/or a lower spacecraft field magnitude pushes the optimal position of the two sensors inwards to get a better signal-to-noise measurement of the spacecraft dipole field component. Stronger higher-order moments of the spacecraft field push the optimal positions of the sensors outwards because of the limitations of using a dipolar field model of Eq. (7). These dependencies were also analytically observed by Neubauer 1975 (see Eqs. (6) and (7)). However, Neubauer’s analysis was restricted to exploring the effect of varying the spacecraft field intensity and non-dipolarity (see Fig. 1 of Neubauer 1975) and did not assess variations in the sensor noise and offsets.

Although the chosen locations of magnetometer sensors on the boom (5.2 m, 6.8 m, and 8.5 m) are not ideal from a gradiometry perspective, the locations do optimize sensor placement in the presence of mechanical constraints and also result in excellent gradiometric performance for various noise scenarios and scaling of the spacecraft field. The approach described in this work allows us to estimate the impact of such constraints on the magnetic field measurements. For example, even if the offset uncertainty is on the order of +/- 0.5 nT (see panel (c) of Fig. 8), the error in the estimated ambient field is not expected to exceed 0.4 nT for the mechanically favorable sensor locations when operating with high instrument flicker noise of $100\ \mathrm{pT}/ \sqrt{\mathrm{Hz}}$ at $1 \mathrm{Hz}$. For comparison, the optimal position of the sensors was determined to be 8.5 m, 7.5 m, and 2.75 m for the same level of noise, which resulted in an estimated ambient field error of less than 0.27 nT. However, changing to this configuration would incur significant cost increases and schedule delays with only a modest increase in 3-sensor gradiometry performance and robustness to outboard sensor failure. The errors in the estimated field are expected to be less than 0.4 nT at the mechanically-optimal position due to the fact that the offsets should be known better than +/- 0.5 nT and the flicker and white noise of the sensors is expected to be significantly less than what is simulated here, likely by a factor of at least five based on noise measurements acquired by the instrument. Additionally, the locations of the current sensor configuration become closer to optimal under more pessimistic spacecraft field scenarios as illustrated in Fig. 8. Therefore, the mechanically optimal sensor locations will be retained for the mission.

## Conclusion

A new magnetic field model of the Europa Clipper spacecraft has been developed and is comprised of 260 different magnetic materials and current-induced field point sources at various locations around the spacecraft. The model is demonstrated to be useful for assessing the magnetic field and its uncertainty at the locations of the fluxgate magnetometer and Faraday cup plasma sensors. Additionally, the model is shown to be useful for visualizing the magnetic field lines associated with the entire spacecraft, which could be used to provide insight into how charged particles may be deflected around the spacecraft and into or away from particle detectors by identifying magnetic mirror points, in addition to visually identifying regions of higher-order magnetic field structure at the locations of the magnetic-field-sensitive sensors. Also shown is the ability to remove spacecraft field from the magnetometer measurements using either a linear or non-linear based gradiometry approach.

Although the present magnetic field model is very mature, there are many magnetic material and current sources on the spacecraft that have yet to be fully characterized magnetically. This characterization will be performed on the ground in the next year before launch and is planned to be described in more detail in a follow-on publication. Additionally, the cruise phase of the mission will be essential for characterization and validation of the spacecraft magnetic field model and also characterizing the AC magnetic field sources on the spacecraft. By meticulously turning on individual subsystems or instruments and switching between different modes, we will monitor the associated fields with the magnetometer suite so they can be removed if on during sensitive measurements. While we do not anticipate any of the magnetic field sources on the spacecraft to directly generate AC field signatures at the key periods (e.g. 11 hour synodic period of Jupiter and 85 hour orbital period of Europa), some of the components may indirectly create field oscillations at these periods due to Clipper’s 6:1 orbital resonance with Europa. For example, the temperature in the vault, where the ECM electronics will reside, is likely to fluctuate with this resonance due to the instruments and subsystems being more active near Europa. The increased current draw during the Europa encounters will result in increased temperature in the vault causing very small periodic changes in the instrument gain. However, because we’ve characterized the instrument’s dependence on electronics unit temperature, we’ll be able to remove the effect of thermal variation during calibration post-processing. The other AC magnetic field source that is of a potential concern are the currents from the solar array, batteries, PCDA, and loops in the harness connecting these components, likely also able to generate magnetic fields with periods associated with the harmonics of Europa’s orbital resonance. Fortunately, we will also be able to back out this effect from the dataset as their field signature will be characterized during the multi-year cruise campaign. These AC field sources will be difficult to removed and therefore will be addressed in more detail in follow-on publication.

## Methods

In this work, we use Matlab for all the data processing and visualizations illustrated in this manuscript. The Europa Clipper spacecraft magnetic field model was captured in a spreadsheet and is available to download as a text file (*Europa_MagneticModel_ConfigF*.*txt*) as supplemental information. Details about how to use the spreadsheet are contained within the document.

For the field line drawing of the spacecraft field in Sect. 4, we use the Euler method. In this method, the algorithm is initialized with a location of interest $\boldsymbol{r}_{n} = \hat{x} x_{n} + \hat{y} y_{n} + \hat{z} z_{n}$, where the magnetic field is calculated via Eq. (1) and defined by $\boldsymbol{B}_{\mathit{SC}} ( \boldsymbol{r}_{n} )= \hat{x} B_{x,n} + \hat{y} B_{y,n} + \hat{z} B_{z,n}$. The location of interest in this case represents the locations in which it is desired to have a passing magnetic field line. The location of the next field sample to be evaluated on the $n +1$ iteration is,

7 $$ \boldsymbol{r}_{n+1} = \boldsymbol{r}_{n} \pm h f \left ( \boldsymbol{r}_{n}, \boldsymbol{B}_{\mathit{SC}, \boldsymbol{r}_{n}} \right ) $$

where $h$ is the step size (small fraction of a meter), $f \left ( \boldsymbol{\cdot} \right )$ is a function which determines the step direction, and ± indicates that two lines are drawn from the initialization location, one in the positive direction and one in the negative direction. (Two field lines are assumed to be needed to connect the north and south poles of a dipole source). Because it is desired to step in a direction that is along the local field direction, the differential step $d \boldsymbol{r} = \hat{x} dx+ \hat{y} d \mathrm{y}+ \hat{z} dz$ should be proportional to the magnetic field, e.g. $\boldsymbol{B}_{\mathit{SC}, \boldsymbol{r}_{n}} \boldsymbol{\mid \mid} \ d \boldsymbol{r}$, where

8 $$ \frac{dx}{B_{x,n}} = \frac{dy}{B_{y,n}} = \frac{dz}{B_{z,n}} = \frac{dr}{\left \vert \boldsymbol{B}_{\mathit{SC}} ( \boldsymbol{r}_{n} ) \right \vert} $$

The location of the next sample, $\boldsymbol{r}_{n+1} = \hat{x} x_{n+1} + \hat{y} y_{n+1} + \hat{z} z_{n+1}$, is computed by,

9a $$ x_{n+1} = x_{n} \pm h \frac{dx}{dr} = x_{n} \pm h \frac{B_{x,n}}{\left \vert \boldsymbol{B}_{\mathit{SC}} ( \boldsymbol{r}_{n} ) \right \vert} $$

9b $$ y_{n+1} = y_{n} \pm h \frac{dy}{dr} = y_{n} \pm h \frac{B_{y,n}}{\left \vert \boldsymbol{B}_{\mathit{SC}} ( \boldsymbol{r}_{n} ) \right \vert} $$

9c $$ z_{n+1} = z_{n} \pm h \frac{dz}{dr} = z_{n} \pm h \frac{B_{z,n}}{\left \vert \boldsymbol{B}_{\mathit{SC}} ( \boldsymbol{r}_{n} ) \right \vert} $$

The locations of the magnetic field line $\boldsymbol{r}_{n+1}$, and their associated magnetic fields $\boldsymbol{B}_{\mathit{SC}} ( \boldsymbol{r}_{n+1} )$, are calculated in this fashion until the field line (1) diverges beyond a configurable distance or (2) exceeds a configurable number of data points so the line does not continue to loop forever in a high flux region.

### Supplementary Information

Below is the link to the electronic supplementary material.

(TXT 19 kB)

## Appendix

Animated visuals are available for download (high resolution .gif and low-resolution .mp4) at the following links:

https://europa.nasa.gov/resources/176/magnetic-field-of-the-europa-clipper-spacecraft/

https://europa.nasa.gov/resources/177/europa-clipper-spacecraft-distortion-of-the-ambient-magnetic-field/.
